# Regionally-Specified Second Trimester Fetal Neural Stem Cells Reveals Differential Neurogenic Programming

**DOI:** 10.1371/journal.pone.0105985

**Published:** 2014-09-02

**Authors:** Yiping Fan, Guillaume Marcy, Eddy S. M. Lee, Steve Rozen, Citra N. Z. Mattar, Simon N. Waddington, Eyleen L. K. Goh, Mahesh Choolani, Jerry K. Y. Chan

**Affiliations:** 1 Department of Reproductive Medicine, KK Women's and Children's Hospital, Singapore, Singapore; 2 Experimental Fetal Medicine Group, Department of Obstetrics and Gynaecology, Yong Loo Lin School of Medicine, National University Health System, Singapore, Singapore; 3 Neuroscience and Behavioral Disorder Program, Duke-NUS Graduate Medical School, Singapore, Singapore; 4 Richard M. Lucas Center for Imaging, Radiology Department, Stanford University, Stanford, California, United States of America; 5 Cancer and Stem Cell Biology Program, Duke-NUS Graduate Medical School, Singapore, Singapore; 6 Gene Transfer Technology Group, Institute for Women's Health, University College London, London, United Kingdom; 7 Faculty of Health Sciences, University of the Witswatersrand, Johannesburg, South Africa; University of Freiburg, Germany

## Abstract

Neural stem/progenitor cells (NSC) have the potential for treatment of a wide range of neurological diseases such as Parkinson Disease and multiple sclerosis. Currently, NSC have been isolated only from hippocampus and subventricular zone (SVZ) of the adult brain. It is not known whether NSC can be found in all parts of the developing mid-trimester central nervous system (CNS) when the brain undergoes massive transformation and growth. Multipotent NSC from the mid-trimester cerebra, thalamus, SVZ, hippocampus, thalamus, cerebellum, brain stem and spinal cord can be derived and propagated as clonal neurospheres with increasing frequencies with increasing gestations. These NSC can undergo multi-lineage differentiation both in vitro and in vivo, and engraft in a developmental murine model. Regionally-derived NSC are phenotypically distinct, with hippocampal NSC having a significantly higher neurogenic potential (53.6%) over other sources (range of 0%–27.5%, p<0.004). Whole genome expression analysis showed differential gene expression between these regionally-derived NSC, which involved the Notch, epidermal growth factor as well as interleukin pathways. We have shown the presence of phenotypically-distinct regionally-derived NSC from the mid-trimester CNS, which may reflect the ontological differences occurring within the CNS. Aside from informing on the role of such cells during fetal growth, they may be useful for different cellular therapy applications.

## Introduction

Neural stem cells (NSC) are multipotent cells found within the central nervous system (CNS) which can give rise to all three neural lineages of neurons, glial and oligodendrocytes [1], [2], [3], [4]. They have gathered significant interest due to the role they play in neural development, as well as their potential for stem cell-based therapy for neurological diseases such as Huntington Disease, amyotrophic lateral sclerosis, Parkinson Disease, multiple sclerosis and stroke among others [5], [6], [7], [8], [9], [10]. Recently, a conditionally immortalised fetal NSC (fNSC) line for the treatment of ischaemic stroke in the United Kingdom has been initiated [11], [12], and a Phase 1 study involving transplantation of fNSC in patients suffering from Pelizaeus-Merzbacher disease showed successful engraftment and donor-derived myelination [13]. The rapid pace of bench-to-bedside research in this field reflects the uniformly dismal prognosis, and the urgent demand of effective treatment for these common debilitating neurological conditions. In addition, sources of neural cells for clinical transplantation have been largely derived from fetal neural tissues, with earlier clinical trials using un-sorted, poorly-characterised neural tissues for the treatment of Parkinson Disease [14], [15]. The identification and characterisation of well-defined human NSC raises the prospect of increasing the efficiency of a cellular transplantation approach for treating different neural injuries, through lineage-specific cellular replacement, the delivery of trophic factors, immune modulation and reduction of inflammation [16], [17], [18]. This approach should also lead to safer well-defined therapeutics.

In the adult human CNS, NSC has been identified only in the subventricular zone (SVZ) and the metabolically active dentate gyrus of hippocampus [1], [3], [19], [20], [21]. In the developing fetus, however, successful isolation of fNSC have been described from many other different regions, including first trimester forebrain, mesencephalon, telencephalon [22], [23], [24], [25], [26] and spinal cord [23], [24], [27], alluding to their developmental role in fetal life. Emerging evidence suggests that fNSC isolated from the different regions of the fetal brain have fundamental differences, such as their immunophenotype, proliferation and differentiation capacity [23], [28], [29], [30], [31]. These differences suggest a regional specification which may be regulated through intrinsic activation of key transcription factors [32], [33], [34], or through the exposure to patterning molecules such as Shh (sonic hedgehog) or FGF (fibroblast growth factor) [35]. In addition, the differences indicated that cells isolated from the respective neurogenic regions retained epigenetic memory of their tissue of derivation [30].

The second trimester CNS undergoes significant changes, with generation and migration of cortical neurons being a key feature [36], coupled with structural changes, including the appearance of the Sylvian fissure and corpus callosum, enlargement at the anterior horns and thinning of inferior and posterior horns of the lateral ventricles [37]. Cellular differentiation during the second trimester is complex, with multiple neuronal subtype arising from several subtypes of progenitors necessary for proper development of the human cerebral cortex [38]. However, to this end, NSC have only been isolated from the SVZ [6], [39] and cerebral cortex [40], [41] of second trimester fetuses. We hypothesised that fNSC derived from the various regions of the second-trimester CNS have different functionalities and neurogenic potential. Here we attempted to isolate NSC from eight different regions of the second trimester CNS, and characterize their ability for clonal propagation and multi-lineage differentiation. By studying regional NSC derived from same donors, we aimed to describe the possible different developmental roles of regional NSC during fetal neurogenesis, and allude to their potential applications in cellular replacement therapy.

## Materials and Methods

Materials are from Gibco-Invitrogen unless otherwise stated.

### Ethics – Samples and Animals

All human tissue collection for research purposes was approved by the Domain Specific Review Board of National University Health System (D06/154). In all cases, patients gave separate written consent for the use of the collected tissue. Gestation was determined by ultrasound measurements of crown-rump length up to 14 weeks gestation and from the bi-parietal diameter between 14–23 weeks gestation. The samples gathered were between gestation weeks of 14^+6^ and 23^+1^ (Table 1). Institutional Animal Care and Use Committee at National University of Singapore and SingHealth approved the use of ICR mice for vivo transplantation for this study.

**Table 1:** Table showing 11 samples used in this study.

Fetal Samples	Gestation (weeks^+days^)	Purpose
1	14^+6^	NS-IC
2	17^+0^	NS-IC/Differentiation
3	20^+0^	NS-IC/Differentiation
4	20^+3^	NS-IC
5	23^+1^	NS-IC
6	18^+0^	Differentiation
7	14^+0^	Microarray
8	18^+0^	Microarray
9	23^+0^	Microarray
10	18^+0^	IUT
11	16^+0^	IUT

NS-IC – Neurosphere Initiating Cultures; IUT – Intrauterine transplantation.

### Preparation of Fetal Neural Tissue

The hippocampus, SVZ, anterior and posterior cerebrum, thalamus, cerebellum, brain stem and spinal cord from the fetal CNS between 14–23 weeks of gestation (n = 11) were isolated and mechanically minced with a scalpel, enzymatically dissociated in 0.25% trypsin for 15 min at 37°C, which was quenched with an equal volume of 40 mg/ml of BSA suspended in Earles' balanced salt solution. The resulting cell suspension was then filtered through 70 µm filters (BD Biosciences, Franklin Lakes, NJ) and washed twice with PBS before enumeration. Viability of cells was determined with 3% acetic acid with methylene blue (StemCell Tech, Canada).

### Neurosphere Culture

For neurospheres initiating assays, cells were plated at a concentration of 3×10^4^ cells per ml of neurosphere medium (1∶1 DMEM: F12 supplemented with 1% N2 supplement, 20 ng/ml hEGF and bFGF (Peprotech, Rocky Hill, NJ), 50 ng/ml of leukemia inhibitory factor (Sigma-Aldrich, St. Louis, MO, USA) and 1X antibiotic/antimycotic in 6 well plates for four weeks. The number of neurospheres with diameter measuring larger than 50 µm as seen under a phase contrast microscope were enumerated in triplicate wells after three weeks of culture.

Subculturing was carried out every two to four weeks, depending on number and size of neurospheres formed. TrypLE Select and mechanical dissociation were used to dissociate the neurospheres into single cells suspension which were then enumerated before plating onto ultra-low attachment 6 well plates (Corning, Cambridge, MA) at 5.5×10^5^ cells/ml and the medium refreshed partially (1∶1) every three days.

Neurospheres to be stained during immunocytochemistry were left to adhere onto poly-lysine coated coverslips for 4 hours at 37°C before fixation with 1∶1 methanol acetone for 5 min at −20°C.

### In vitro Differentiation of fNSC

Neurospheres were dissociated as above and cultured in differentiation medium (DMEM-F12 with 1% N2 supplement, 1% fetal bovine serum and 1X antibiotic/antimycotic on coverslips coated with poly-L-ornithine for one week before washing with PBS and fixed with 1∶1 methanol acetone for 5 min at −20°C for analysis.

### Immunocytochemistry

Fixed cells were incubated in protein blocking agent (ThermoElectron, United States) for 60 min at room temperature. The samples were then incubated with primary antibody: anti-BIII-Tubulin) mouse monoclonal (1∶100, Sigma-Aldrich), anti-GFAP (glial fibrillary acidic protein) rabbit polyclonal (1∶400; Sigma-Aldrich), anti-PDGFRα (platelet-derived growth factor receptor alpha) rabbit monoclonal (1∶100; Millipore, MA, United States), anti-nestin mouse monoclonal (1∶100; Millipore), anti-SOX1 rabbit monoclonal (1;100, Millipore), anti-SOX2 mouse monoclonal (1;100, Millipore), anti-Ncadherin mouse monoclonal antibody (1∶100,Millipore), anti-S100B mouse monoclonal (1∶100, Calbiochem, Darmstadt, Germany) and anti-NeuN rabbit monoclonal (1∶100, Millipore) for 60 min at room temperature. After washing the slides with PBS (phosphate buffered saline) twice, they were incubated for 30 min at room temperature in the dark with 594 AlexaFluor-labelled goat anti-mouse antibody: PBS (1∶400) and 488 AlexaFluor-labelled goat anti-rabbit antibody (1∶400) (Molecular Probes, CA, United States), washed twice with PBS before being set with mounting medium containing DAPI (Vector Laboratories, CA, United States). Slides were visualised with a laser confocal fluorescence microscope (Fluoview FV1000, Olympus, Japan). All secondary-only controls were negative for staining (data not shown). The percentage of neurons and astrocytes generated were determined by counting the numbers of BIII-tubulin-positive or GFAP-positive cells respectively, as a percentage of DAPI-positive nuclei in at least five random low-powered-fields, with a median number of 236 cells counted per field (Range: 100–360, n = 3 different fetal samples). Data are expressed as mean ± SEM unless otherwise specified.

### Virus Preparation and Lentiviral Transduction of fNSC

fNSC were stably transduced with a lentiviral vector (FUGW) encoding the green fluorescent protein (GFP) reporter gene driven by the human ubiquitin-C promoter, and pseudotyped with the G glycoprotein of the vesicular stomatitis virus envelope. Lentiviruses were produced as previously described [42]. Briefly, HEK293T cells were triple transfected with vector (FUGW-EGFP), core and envelope plasmid through a standard calcium phosphate precipitation protocol. The supernatant was harvested at 48 and 72 hours, pooled and concentrated at 90,000 g for 90 min at 4°C. fNSC were then exposed to the virus for 24 hours followed by three days of culture. GFP was detected at day 4 with a transduction efficiency of 90%.

### Intrauterine fNSC Transplantation

Intrauterine transplantation was performed as previously described [43]. Briefly, time-mated ICR females were anesthetized and uterine horns exteriorised. 1×10^5^ hippocampal-derived EGFP-labelled-fNSC in 1 µl of PBS were injected into the lateral ventricle of each embryo at embryonic day (E) 13 with a micro glass capillary, and the embryos replaced and abdomen closed. Transplanted dams were allowed to litter naturally.

### Immunohistochemistry

Immunohistochemistry was performed as previously described [44]. Briefly, injected animals were perfused at different time points with 4% paraformaldehyde (PFA). The brains were removed and immersed in 4% PFA overnight at 4°C and then equilibrated in 30% sucrose. Entire brains were processed in sagittal 40 microns microtome sections. Sections which were positive with GFP-labelled cells were blocked with 5% donkey serum, permeabilized with 0.25% Triton X in TBS for 30 minutes before application of primary antibodies of rabbit anti-GFAP (Dako, Glostrup, Denmark), anti-PDGFRα (Millipore), goat anti-doublecortin (Santa Cruz, CA, USA), mouse anti-human nestin (Millipore) and anti-human nuclei (Millipore) at dilutions of 1∶100 to 1∶500 for overnight incubation at 4°C. Incubations with secondary antibody (1∶500) of 647 donkey anti-rabbit or 647 donkey anti-goat with 555 donkey anti-mouse for one hour at room temperature were performed, followed by a five minute staining with DAPI (Millipore), before sections were mounted on slides with mounting medium. The staining was viewed using Zeiss LSM 710 confocal system (Carl Zeiss Pte Ltd, Singapore) at 63X magnification.

### Microarray

Total RNA was extracted using Trizol from three clinical samples at 14, 18 and 23 weeks gestation. Ten micrograms of total RNA were used to generate labelled cRNA and hybridised to Human Genome U133 Plus 2.0 arrays (Affymetrix, CA, USA, http://www.affymetrix.com).

**Figure 1:**
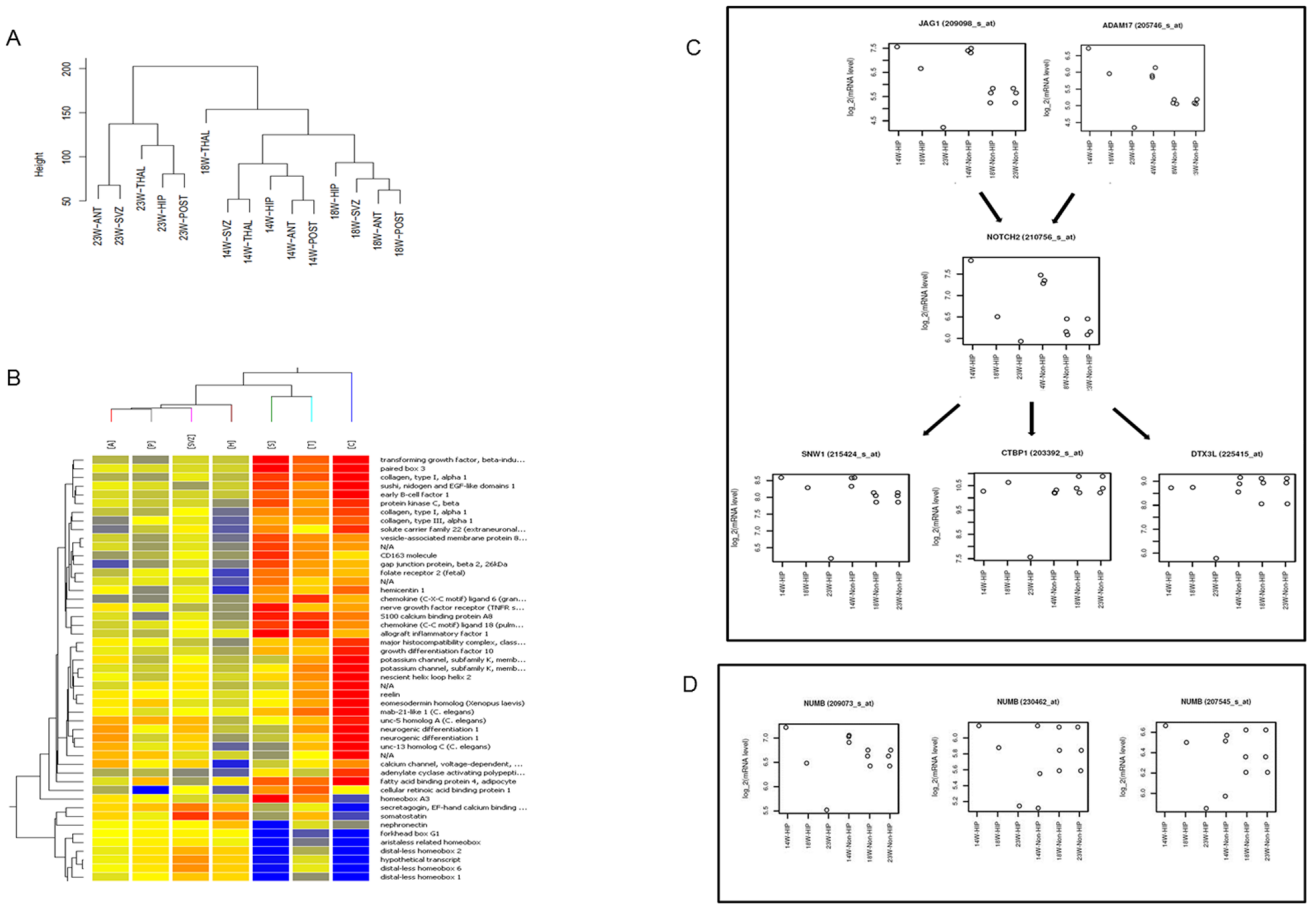
Microarray analysis of RNA extracted from neurospheres derived from different regions of mid trimester fetal brain. Hierarchical clustering demonstrates that gestational differences are more apparent that regional differences in terms of gene expression (A). Heatmap showing the clustering of the neural regions based on the top 50 genes that are differentially expressed between the hippocampus and the non-hippocampal regions (B). Expression of specific genes involved in Notch signaling pathway in the neurospheres cultured from the Hippocampus versus non-hippocampal (A, P and SVZ) (C). Levels of expression of the 3 probes for NUMB by the neurospheres cultured from the hippocampal and non-hippocampal regions (A, P and SVZ) (D). Legend: Anterior[A], Posterior[P], Subventricular zone[SVZ], Hippocampus[H], Brain Stem and Spinal Cord[S], Thalamus[T], Cerebellum[C].

For Fig. 1A, C and D, we used the BioConductor (http://www.bioconductor.org/) ‘affy’ package to get the Robust Multichip Average expression measures (function ‘rma”) and MAS5 ‘A’, ‘M’, and ‘P’ assessments (function ‘mas5calls’, ‘A’ denotes absent expression). We removed probe sets for which MAS5 estimated absent expression in every sample and probe sets with coefficient of variation (standard deviation divided by mean) ≤0.1. The distance between samples using the R dist function with default parameters were computed and hierarchical clustering using the R hclust function was plotted.

GeneSpring GX 11.5.1 (Agilent Technologies, Palo Alto, CA, http://www.agilent.com) was also used to identify differentially-expressed genes (top 50 as shown in Fig. 1B), from which significant pathways were identified (Table 2). The raw signals on each chip were first shifted to the 75^th^ percentile of the chip itself. Thereafter, the median of each gene across samples is calculated and subtracted from each chip's signals to give normalised signals, from which the results are analysed and compared. Of the 54,675 probes on the chip, 40,069 are flagged present or marginally present in one or more of the chips. Of the 40,069 probes, 17,498 probes were at least two fold differentially expressedin the hippocampal sample when compared with the corresponding probes in other samples.

**Table 2:** Table showing the 11 pathways matched with the probes that are ≥2 fold differentially expressed in the hippocampus compared to the other regions.

Pathway	Number of Entities	Matched with Technology	Matched with Entity List	p-Value
Androgen Receptor	98	93	51	0.018
BCR	148	135	77	0.002
EGFR1	181	176	101	>0.001
IL2	72	58	34	0.016
IL4	56	48	31	0.001
IL5	39	33	21	0.013
IL-7	16	16	11	0.034
Kit Receptor	70	64	35	0.029
NOTCH	93	74	44	0.003
TCR	140	126	64	0.038
Atrazine degradation	20	8	8	0.006

### Real-Time Polymerase Chain Reaction

Real time polymerase chain reaction (PCR) were performed in triplicate, in 25 ul: 10 µl cDNA, 12.5 µl TaqMan Universal PCR Master Mix (Applied Biosystems, CA, USA, http://www.appliedbiosystems.com), and 2.5 µl primer working solution. Thermal cycle conditions were 96°C for 2 minutes, then 40 cycles at 96°C for 10 seconds and 60°C for 30 seconds. Amplifications were monitored with the ABI Prism 7000 Sequence Detection System (Applied Biosystems). Results were normalized against the housekeeping gene β-actin III, and relative gene expression was analyzed with the 2^-ddCt^ method. Primers used: NUMB (5′GGCCCACCAATATTCCAATC′3 and 5′ GTGGCGCTTGAGTTGGTC′3), β-actin (5′ TGAC GGGGTCACCCACACTGTGCCCATCTA′ 3 and 5′ CTAGAAGCATTTGCGGTGGACGATGGAG GG′ 3), NOTCH (5′TCGGCAGACTGGTGACTTC′3 and 5′ACAGGTGCTCCCTTCAAAAC′3), JAGGED1 (5′GAATGGCAACAAAACTTGCAT′3 and 5′AGCCTTGTCGGCAAATAGC′3), EMX1 (5′GAGACGCAGGTGAAGGTGT′3 and 5′GTTGATGTGATGGGAGCCCT′3) and HoxB8 (5′TGGAGCTGGAGAAGGAGTTC′3 and 5′CTCCTCCTGCTCGCATTT′3).

### Statistical Analysis

Statistical comparisons between the various anatomical regions were performed using the Kruskal-Wallis test with further analysis using Dunn's Multiple Comparison's Test. A p-value<0.05 was considered to be statistically significant.

## Results

### Neural Stem/Progenitor Cells in Various Regions of the Second Trimester Fetal Brain

**Figure 2:**
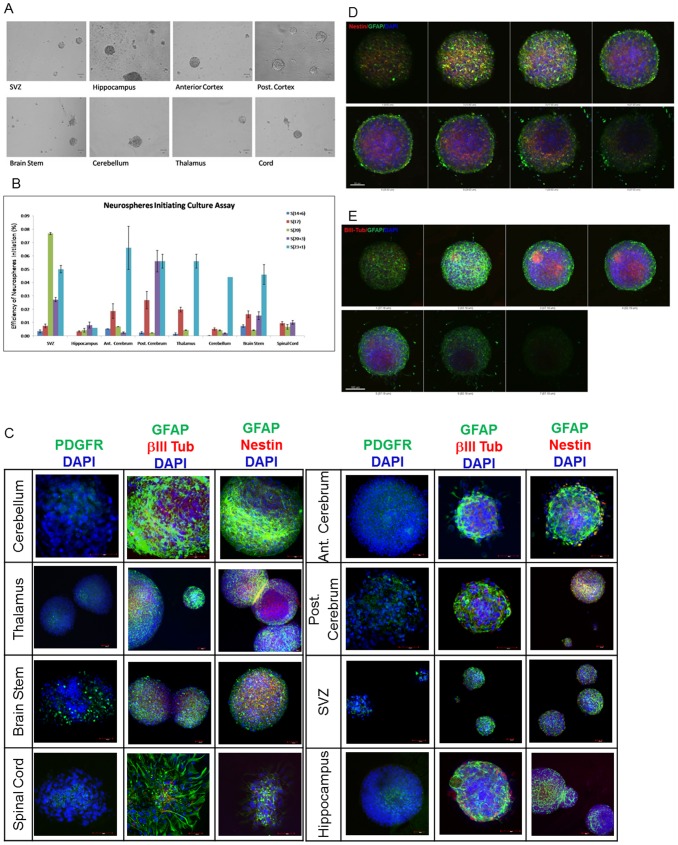
Characterisation of growth properties. Spheres derived from the various regions were morphologically similar (A), Neurospheres initiating efficiency demonstrates an increasing trend with increasing gestation (B). Regional neurospheres in serum-free medium showed presence of all three neural lineages of glial (GFAP), oligodendrocyte (PDGFRα) and neurons (BIII-tubulin), and Nestin (C). Cross sectional view of a neurosphere showing predominant GFAP staining in the periphery of the sphere, with Nestin and BIII-tubulin located within the core of the sphere (D–E).

Neurospheres can be seen emerging after a week in culture from all eight regions examined (SVZ, Hippocampus, Anterior Cortex, Posterior Cortex, Brain Stem, Cerebellum, Thalamus and Spinal Cord), and from all seven donors (14–23 weeks gestation) examined. The Thalamus of S(20+3) and the Spinal Cord of S(14+6) and S(23+1) did not yield sufficient cell numbers for the assay. Morphologically, the neurospheres were identified by their phase-bright appearance and smooth well-defined cell membranes around the spherical structures. Microscopically, there is little difference in the physical appearance of these regionally-derived neurospheres from the second trimester fetal brain (Fig. 2A).

The efficiency of neurosphere generation ranged between 0.002 to 0.070% (n = 5, 14^+6^ to 23^+1^ weeks^+days^), with the highest frequencies of neurosphere-initiating capacity (NS-IC) found in the SVZ and cerebrum, and the lowest in the spinal cord and hippocampus (Fig. 2B, Table S1). We observed the lowest NS-IC in the hippocampus (0.002%±0.000%) and the highest in the brain stem (0.0074%±0.009%) from the sample at the lowest gestational age (14^+6^ weeks), and from the fetus at the highest gestational age investigated (23^+1^ weeks), we found the lowest NS-IC in the hippocampus (0.006%±0.000%), with the anterior cerebrum with the highest NS-IC (0.066%±0.016%) (Fig. 2B). An increasing trend for NS-IC was observed between 14 weeks and 23 weeks, suggesting that the pool of NSC increases throughout the second trimester (Fig. 2B). We found significant differences of the NS-IC between the different regions from 14 to 20 weeks gestation (p<0.05), but not for the later gestation of 23^+1^ weeks (Table 3).

**Table 3:** P-values determined by Kruskal Wallis Test, when comparing for region specific differences in NS-IC within each gestational age.

Gestation(weeks^+days^)	P-Value
14^+6^	0.0129
17^+0^	0.003
20^+0^	0.003
20^+3^	0.010
23+^1^	0.113

All regionally-derived neurospheres in culture expressed markers of all three neural lineages, being positive for β-tubulin isotype III (BIII-Tubulin), GFAP and PDGFRα, representing neuronal, glial and oligodendrocytic lineages respectively (Fig. 2C). Nestin positive cells were predominantly located within the centre of the neurospheres, with spontaneous glial differentiation found towards the periphery of the sphere (Fig. 2D). A similar pattern of staining was observed for neurospheres double-stained for both GFAP and BIII-Tubulin (Fig. 2E), where GFAP-positive cells were found along the periphery and BIII-Tubulin^-^positive cells located within the centre of the spheres.

### Differentiation Potentials of Regionally-derived NSC

**Figure 3:**
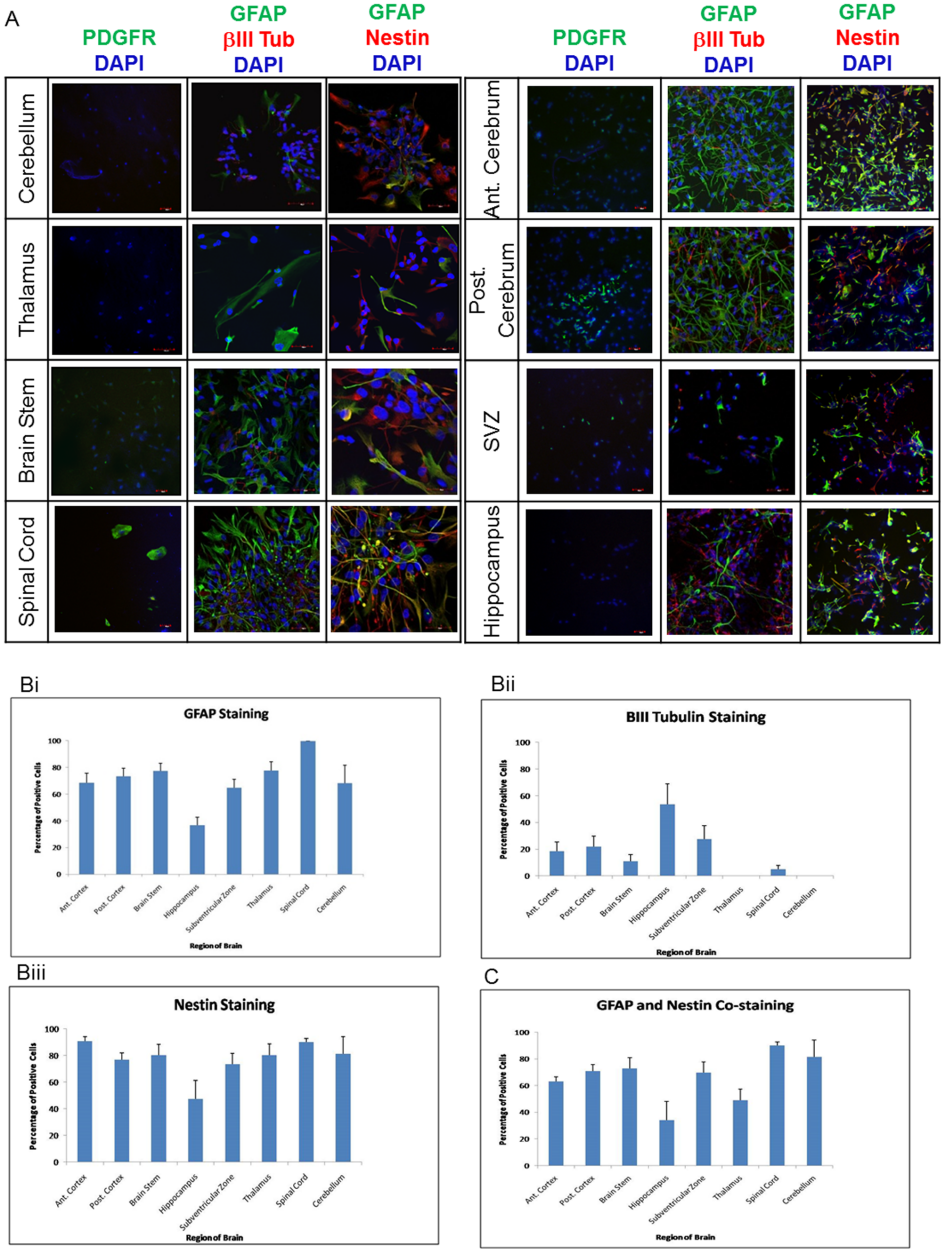
Differentiating potential of cells in neurospheres derived from different regions of mid-trimester fetal brain. Trypsinised dissociated cells from regional-neurospheres were placed in differentiation medium over poly-L-lysine slides and stained for GFAP, BIII-Tubulin, PDGFRα and nestin (A). Graphical representation of the staining profile for GFAP, BIII-Tubulin and nestin across the various regions of the fetal brain (Bi, Bii and Biii respectively) and of the co-staining of GFAP and nestin (C).

Next, we investigated the differentiation capacity of the regional-fNSC at passage 0 (n = 3 samples at 17, 18 and 20 weeks gestation, Table 1) from all eight regions by plating cells from dissociated neurospheres onto poly-L-ornithine cover slips, withdrawal of growth factors and the addition of serum for over a week. Dissociated neurospheres adhered and migrated across the coverslips, forming networks of intertwined cells after a week of culture (Fig. 3A). We found a range of 36.6% to 99.7% cells staining positive for GFAP (Fig. 3Bi), 0% to 53.6% staining for BIII-Tubulin (Fig. 3Bii), 77.0% to 90.7% of staining for nestin (Fig. 3Biii), with minimal staining for oligodendrocytic marker PDGFRα. In addition, NeuN and S100b were also positively stained in the cultures (Fig. S1). No gestational related differences nor trends were observed across the three samples. There were significant differences in the differentiation capacity of regionally-derived fNSC into glial (p<0.001) and neural lineages (BIII-Tubulin, p<0.004), but not nestin (p = 0.06). In particular, the highest neuronal differentiation was achieved in hippocampal fNSC (53.6±15.4%). We did not observe any neuronal differentiation seen in fNSC derived from the thalamus and cerebellum despite the presence of BIII-tubulin-positive cells within pre-differentiation thalamic and cerebellar-neurospheres (Fig. 2C). Co-staining of GFAP with nestin was found in all eight regionally derived samples, with the lowest observed in the hippocampus (34.1±10.0%), and highest in spinal cord (90.1±2.7%) (Fig. 3C). As co-expression of GFAP and Nestin cells suggest their radial-glial lineage, we confirmed through positive staining of Sox1, Sox2 and N-Cadherin (Fig. S1).

We also found the presence of GFAP and BIII-tubulin double positive cells in anterior (5.2±4.2%) and posterior (3.4±3.4%) cerebra, brain stem (6.9±5.1%), SVZ (3.5±3.3%) and spinal cord (21.6±15.2%) but not hippocampus, thalamus and cerebellum.

### Engraftment of Injected NSC in Mice

**Figure 4:**
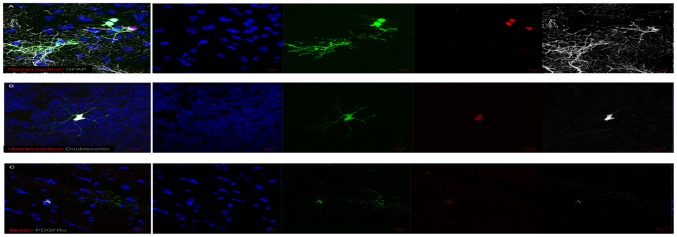
Immunohistochemistry for neuronal markers. Positive co-staining of GFAP (A), doublecortin (B), PDGFRα and nestin (C) were observed on GFP-labelled human cells. Staining for human nucleus (A, B) also confirm the GFP-labelling of the human cells. Bar: 10 µm.

Next, we explored the ability of hippocampal-derived fNSC to engraft into the developing mouse brain. Transplanted pups (n = 5) were allowed to litter, and analysed 6 weeks after surgery. We found the presence of GFP-positive cells in 4 out of the 5 pups (80%). As shown in Fig. 4, transplanted GFP-positive cells are found in clusters, with some having migrated into the surrounding brain tissue. Incorporated GFP cells displayed the human nuclear marker, the neuronal marker doublecortin, the glial marker GFAP and the oligodendrocyte marker PDGFRα (Fig. 4A, B and C). This data demonstrated the differentiation and migration potential of fNSC in vivo.

#### Genome-wide Gene Expression

Given the comparatively higher neurogenic potential of hippocampal-fNSC over other regionally-derived fNSC (Fig. 3Bi and ii), we hypothesised that there are intrinsic differences in gene regulation network based upon the region of derivation. For this purpose we examined the global gene expression of regionally-derived fNSC from primary neurospheres cultured in expansion medium. We filtered for genes that are significantly overexpressed or underexpressed by at least twofold in the hippocampus compared to the other regions. The 17,498 probes found in the manner were organised into established pathways on Biopax to identify 11 pathways (Table 2), one of which was the Notch signalling pathway associated with self-renewal of NSC.

Hierarchical clustering showed gestational age to be more closely clustered than regional tissue of derivation (Fig. 1A). Next, we interrogated the differential expression exhibited by neurospheres of the hippocampus compared to the other regions in two samples (14 and 18 weeks). The normalised values of four region-specific genes for the regional fNSC were derived in order to ascertain the origin of the regional-fNSC. The values of the probe of a forebrain marker EMX1, known to be expressed in the developing cerebral cortex [45] was highest in the anterior and posterior cerebra-derived fNSC. A midbrain marker, EN1, expressed in cerebellum [46], [47], was demonstrated to have the highest value in the cerebellum-fNSC. In the same manner, HoxB6 and HoxB8, both of which are spinal cord markers [48], , were most highly expressed in the spinal cord fNSC (Fig. S2A), further supporting the regional identity of these fNSC. These microarray results were corroborated by qPCR (Fig. S2B).

Hierarchical clustering analysis of the top 50 differentially-regulated genes showed that the global cell expression profile of hippocampus was overall similar to anterior, posterior cerebra and SVZ and more different compared to spinal cord, thalamic and cerebellar fNSC. We noted that the eventual result of the clustering reveals a similar result to that of anatomical regions, suggesting regionalisation that is reflected in the differential expression of the top 50 genes significantly different in the hippocampus when compared to the other regions. A heatmap of the top 50 genes was developed to visualise the relative expression of clustered genes (Fig. 1B).

Probing further, we looked at specific genes in the Notch pathway which are differentially expressed. In this analysis, we looked at the effect of gestation on the expression of six genes in the Notch Pathway in Hippocampal fNSC (HIP) and non-hippocampal fNSC from the Anterior and Posterior Cortex, and SVZ (Non-HIP). Non-HIP NSC have a similar expression level of all six genes; JAG1 (Jagged1), ADAM17, NOTCH2, SNW1, CTBP1 (C-terminal binding protein of adenovirus E1A) and DTX3L (deltex 3-like). This is shown by the close proximity of each of the circles, representing each sample, in these non-hippocampal regions (Fig. 1C). Expression of the upstream genes JAG1 and ADAM17, and NOTCH2 were higher at 14 weeks gestation than at later gestations, while expression of the downstream genes, SNW1, CTBP1 and DTX3L were similar across all gestations (Fig. 1C). On the other hand, HIP had high expression of all six genes at 14 and 18 weeks gestation, but much lower expression levels at 23 weeks (Fig. 1C). In the case of NUMB, an inhibitor of Notch, we noted a drop in expression levels with increasing gestation in the hippocampal region, while the expression levels remain similar in the non-hippocampal regions (Fig. 1D). We further confirmed this finding through qPCR for selected genes, with HIP fNSC expressing higher levels of NUMB (4.8 fold), JAG1 (8.9 fold) and NOTCH2 (33.8 fold) (Fig S2C).

## Discussion

Fetal neural development is a tightly regulated process whereby differentiation of regions leads to an integrated CNS. While fNSC has been derived in the second trimester CNS from the SVZ, cortical tissue and SC [28], [31], [39], it is currently not known whether other regions of the brain are capable of giving rise to putative fNSC, and whether they are influenced by gestational effects. Here we demonstrate that in addition to the three known regions, the second trimester hippocampus, thalamus, cerebellum and brain stem are capable of giving rise to multipotent neurospheres with increasing frequencies as gestation advances. By working with a number of regionally-derived fNSC from the same donors, we provided evidence that there are striking differences in their capacity for lineage-specific differentiation, from the highly neurogenic hippocampal fNSC, to the other regions which differentiates down the glial lineage predominantly. Furthermore, transcriptomic studies highlight the regional differences and the importance of the Notch pathway in regulating these observations.

In this paper, we confirmed through the successful derivation of neurospheres, the presence of NSC from all anatomical regions of the second-trimester CNS. We find that the cerebrum contained the highest frequencies of NS-IC, which may reflect the massive growth of the cerebrum during the second trimester of fetal life. This is in contrast with findings in the adult post-natal brain, where NSC has only been found in the metabolically active SVZ and hippocampus. In addition, we document a general increase in NS-IC efficiency with increasing gestation between 14 to 23 weeks. This contrasts with a fall in NS-IC frequencies between E12 (10%) and the immediate postnatal rat brain. between E12 and P1 rats [50], [51]. Using limiting dilution assays on minced fetal CNS tissues, Uchida established the NS-IC in second trimester human fetal brain to be 1 out of 880 i.e. 0.11% [39] which is two folds higher than our own observations. This difference could be due to the different protocol of harvesting; from the tissues/region harvested to the digestion protocol as well as the difference in the gestational age of the samples used and/or a combination of both. Significant differences in the neurosphere forming ability of the different anatomical regions was observed from samples of 14 to 20 weeks (**Table 3**), illustrating the differences between the regional-fNSC. We did not observe any differences in NS-IC of regional-fNSC in the 23 week sample on Kruskal-Wallis analysis of variance (Table 3). This may be due in part due to the larger standard deviation, particularly in ant. cerebrum and the collectively high values noticed across all regions except hippocampus.

By using sectional analysis of stained neurospheres, we found the more primitive nestin-positive cells to be located at the core of the neurospheres, with glial differentiation being prominently observed at the periphery. Co-staining of GFAP with BIII-Tubulin or nestin had been observed in fetal derived ependymal and radial glia cells, contributing to their identity as multipotent cells [52], [53]. We validated this co-staining by staining for N-cadherin, Sox 1 and Sox 2 which has been described as a radial glial cell marker [54], [55], [56], [57]. This similar co-staining at varying proportions in our study further suggests the high level of heterogeneity in terms of the state of maturity of the cells. This is in keeping with Suslov and colleague's report through molecular phenotyping of individual neurospheres which demonstrated a heterogenous cell population at various stages of lineage commitment, ranging from the rare primitive NSC, estimated at 1% of all cells, to the other 99% of lineage-restricted progenitors and terminally-differentiated neural cell types of neurones, glial cells and oligodendrocytes [58], [59]. Thus it is likely that the more quiescent true NSC reside within the core of the neurosphere, with rapidly proliferating glial cells situated at the periphery [59], [60].

Both spatial and temporal developmental signals contributing to the patterning and regionalisation of NSC are extremely important as they can help identify essential mediators of stem cell renewal, and genes that determine the production of the various neural lineages [3]. This suggests that regional NSCs have different functional properties which may play a part in their potential for cellular therapy [3]. It is therefore of interest to further evaluate if neurospheres from any particular region are more advantageous for cellular therapy in different disease paradigms, for instance neurospheres derived from hippocampus for neurological disease like Parkinson and Huntington, whereas spinal cord-derived neurospheres for cellular therapy of spinal cord injury. This is in keeping with the findings of Ostenfeld et al, where they found a smaller quantity of larger neurons with longer processes produced from neurospheres derived from the hind brain as compared to those derived from the cortical/striatal regions [30].

We observed lineage specific staining demonstrating the presence of all three neural lineages in the regional neurospheres. Interestingly, no staining for BIII-tubulin was observed from the serum-induced differentiation of the thalamic and cerebellar-derived fNSC. It has been shown that NSC in the CNS undergo neurogenesis forming neurons first before undergoing gliogenesis forming glia cells [61], [62]. Our observations of the temporal manner of lineage staining in neurospheres may reflect the neurogenic phase in the thalamic and cerebellar neurospheres where BIII-tubulin expression is lost during subsequent serum-induced differentiation, during which the gliogenic phase has been initiated. The hippocampus is one of the only two regions in the adult human brain where active neurogenesis occurs, and is also the region responsible for the processing of information when multiple stimuli are involved [63], [64]. Here we show that this phenomenon may be pre-dated by the presence of NSC with particularly high neurogenic potential as early as 14 weeks of gestation. While staining for PDGFRα is minimal, we do observe a difference in the staining pattern. The positive staining in the differentiated cells from the spinal cord appears larger than the other regions, which appears more spindle-shaped (Fig. 3A. These morphological differences seen could be the result of differing cell-cell interactions, as well as the relative density at which the cells had been growing in. We believe this is dependent on the morphology of the cells and their interaction with the neighbouring cells, in which case, those from the spinal cord have a wider area to grow in culture, with fewer cell-cell interactions taking place, which may have resulted in their larger morphological appearance. Although there are morphological differences, their expression of PDGFRα indicates their oligodendrocyte progenitor origins.

Intrauterine transplantation of the human fetal stem/progenitors allowed a follow up of the injected human cells without initiating an immune response from these immunocompetent mice while the access to a large neuroepithelium in a neurologically-enriched environment without immunological response allowed a follow up of the injected human cells. Moreover, transplantation of the human fNSC into the developing mouse brain is a valuable model to study their differentiation potential. Similar to the observations for in vitro differentiation, the fNSC are able to differentiate into the three neural lineages between four to eight weeks. A study comparing the in vivo differentiation potential of the regional fNSC could further define the potentially different capabilities and functional roles of the regional fNSC.

By studying global-gene expression data of fNSC, we observed that gestational age effect of neurospheres clustered more tightly together than do similar regional-NSC of different gestational age through hierarchical clustering. Thus, the particular gestational age of the NSC source may be an important consideration in selection of cells for cellular therapy, which has not been a central consideration to date. Using the top 50 differentially expressed genes, we found similar clustering of regional fNSC to be dictated with proximity of anatomical regions, with the hippocampus clustering more closely to anterior, posterior cerebra and SVZ than to the cerebellum and spinal cord. Aside from the Notch signaling pathway, the EGFR (epidermal growth factor receptor) and interleukin pathways were also identified as being differentially regulated between the hippocampal and other regionally-derived NSC. An upregulation of Notch signaling is necessary to upkeep the undifferentiated ‘stem’ state of NSC [65]. By comparing the hippocampal to the non-hippocampal NSC from 14, 18 and 23 week samples, we find a down-regulation of this pathway with increasing gestation only in the hippocampal NSC, but not in the Anterior and Posterior Cortex, and SVZ-NSC. The lowered expressions of Notch at the later gestational age are reflective of the in vitro system with the NSCs ‘activated’ from the quiescent state to enter the proliferating and differentiating process. Thus, the decreased expression of the genes involved in the Notch signaling pathway in hippocampal NSC may have contributed to the more robust neuronal differentiation observed. This is consistent with recent findings where neurospheres derived from mice constitutively expressing of Notch2 exhibit a reduced neuronal differentiation accompanied with increased tendency for astrocytic differentiation [66]. Falk et al has also demonstrated with the use of anti-Notch antibodies that differentiation of NSC towards a neuronal fate is enhanced when the Notch signaling pathway is downregulated [67]. The lower expressions of Notch pathway associated genes is also accompanied by a corresponding lowered expression of NUMB, an adaptor protein that is an inhibitor of Notch signaling [68]. As NUMB plays an integral role in the establishment of asymmetric cell divisions [68], its reduced expression may lead to the hippocampal-derived NSC undergoing symmetric divisions, thereby sustaining a reserved pool of neural stem cells that are brought forward to adulthood.

The activation of the EGF pathway is crucial for proper development of astrocytes in the developing CNS [69], with over-expression of EGF receptors in NSC resulting in astrocytic differentiation. We have found that a smaller proportion of hippocampal-derived NSC differentiated into glial cells as compared to the other regionally-derived NSCs and it is reflected by the significantly different expressions of pathway associated genes in the hippocampus as compared to the non-hippocampal regions (Table 2). This is also concordant with Sun et al who demonstrated that delivery of EGF intraventricularly into a rat model of post-traumatic brain injury resulted in preferential astrocytic differentiation [70]. Interestingly, a number of inflammatory pathways were flagged as well (Table 2). Cytokines play crucial roles in shaping neural plasticity, differentiation of neuronal cells and formation of memory. Interleukin (IL) 2 is known to promote neurite extension [71], neuronal survival [72], and in promoting the proliferation and maturation of oligodendrocytes [73], [74], while IL5 and IL7 has been implicated in neuronal differentiation and survival as well as neurite outgrowth [75]. As such, it is not surprising that expression of genes associated with the four IL pathways were identified to be significantly different in the hippocampus as compared to the other regions of the brain. This also suggests that inflammation pathways may exist as key steps leading to the differences and consequentially, the different functional roles of cells in the various anatomical regions.

## Conclusions

We have shown NSC can be derived from all regions of the second trimester CNS, beyond just the SVZ, cerebral cortex and spinal cord [28], [39]. While neurospheres generated by the regionally-derived NSC appear largely similar morphologically, we found significant differences between the regions in terms of neurospheres initiating assays, differentiation potential and expression of genes associated with several pathways, including the NOTCH, EGF and inflammation-linked pathways. Further comparisons between the regionally-derived NSC could shed more light on understanding of their role during ontogeny and their potential for cellular therapeutics. In addition, we propose that these regionally-derived NSC could serve as in vitro models in understanding development in the brain, which can be tackled by correlating genome-wide analysis of pathways and specific genes associated with development with gestational age.

## Supporting Information

Figure S1 **Differentiating potential of cells in neurospheres derived from different regions of mid-trimester fetal brain.** Trypsinised dissociated cells from regional-neurospheres were placed in differentiation medium over poly-L-lysine slides and stained for S100B, Sox1 (white arrows), Sox2, NeuN (white arrows) and N-cadherin.
(TIF)

Figure S2 **Expression levels of genes in different regions of the brain.** Gene expression of region specific genes, EMX1 (cortex), EN1 (cerebellar), HoxB6 and HoxB8 (spinal cord) from the microarray (A) with EMX1(Bi) and HoxB8(Bii) corroborated by qPCR. Gene expression of numb, Jagged1 and notch2 in RNA derived from hippocampal and non-hippocampal regions by qPCR (C). The level of expression of numb (RQ:4.8), Jagged 1(RQ:8.91) and notch 2 (RQ:33.8) are higher in the non-hippocampal region.
(TIF)

Table S1 **Table showing the median and range of neurospheres formed per million of regionally-derived cells seeded.**
(DOCX)
