# Supplementary figures and images for: Regionally-Specified Second Trimester Fetal Neural Stem Cells Reveals Differential Neurogenic Programming
Source: PLoS One. 2014 Sep 2;9(9):e105985. doi: 10.1371/journal.pone.0105985 (PMC4152177; doi:10.1371/journal.pone.0105985)

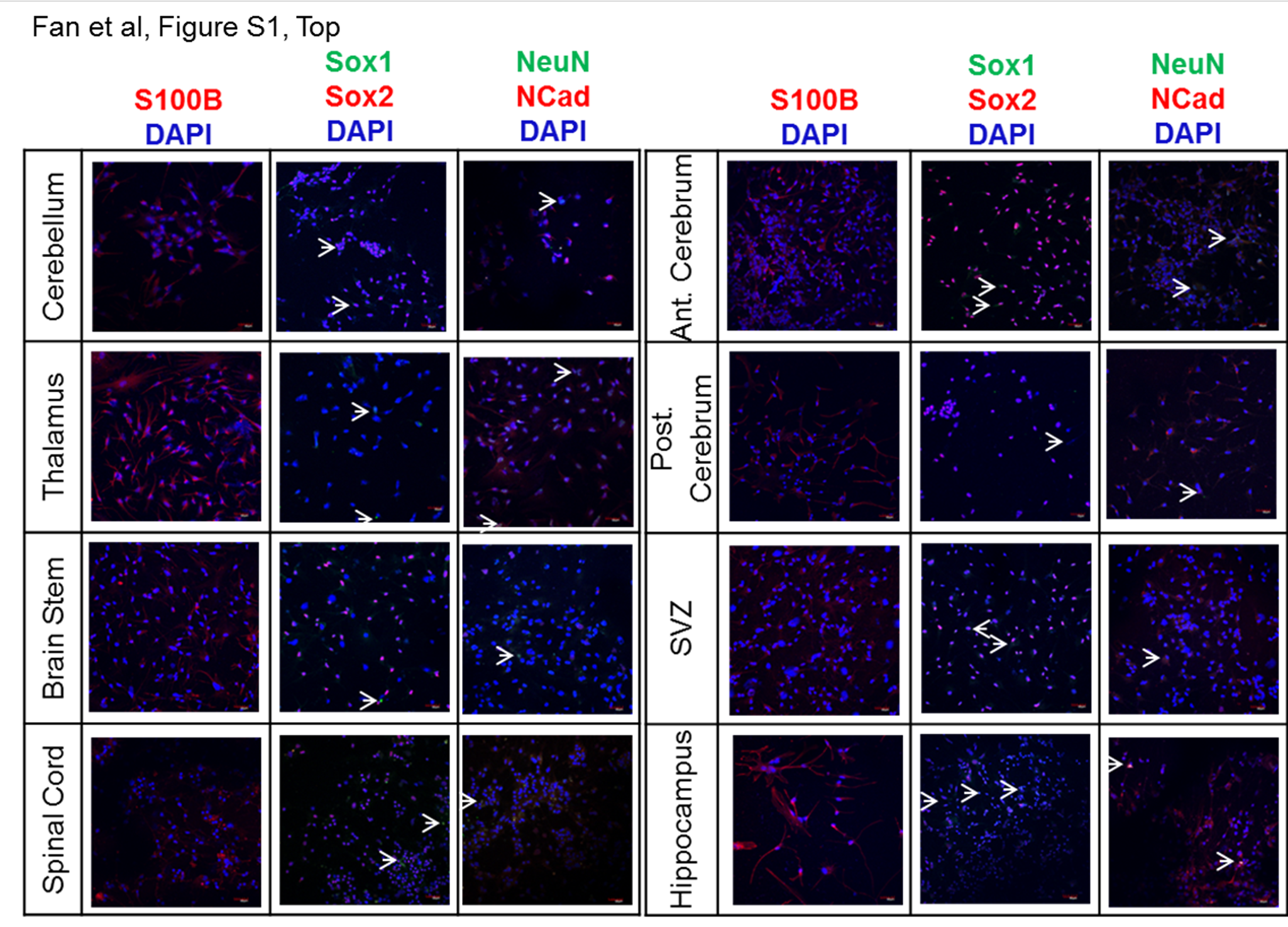

Supplement: Figure S1 — Differentiating potential of cells in neurospheres derived from different regions of mid-trimester fetal brain. Trypsinised dissociated cells from regional-neurospheres were placed in differentiation medium over poly-L-lysine slides and stained for S100B, Sox1 (white arrows), Sox2, NeuN (white arrows) and N-cadherin. (TIF) [file pone.0105985.s001.tif]

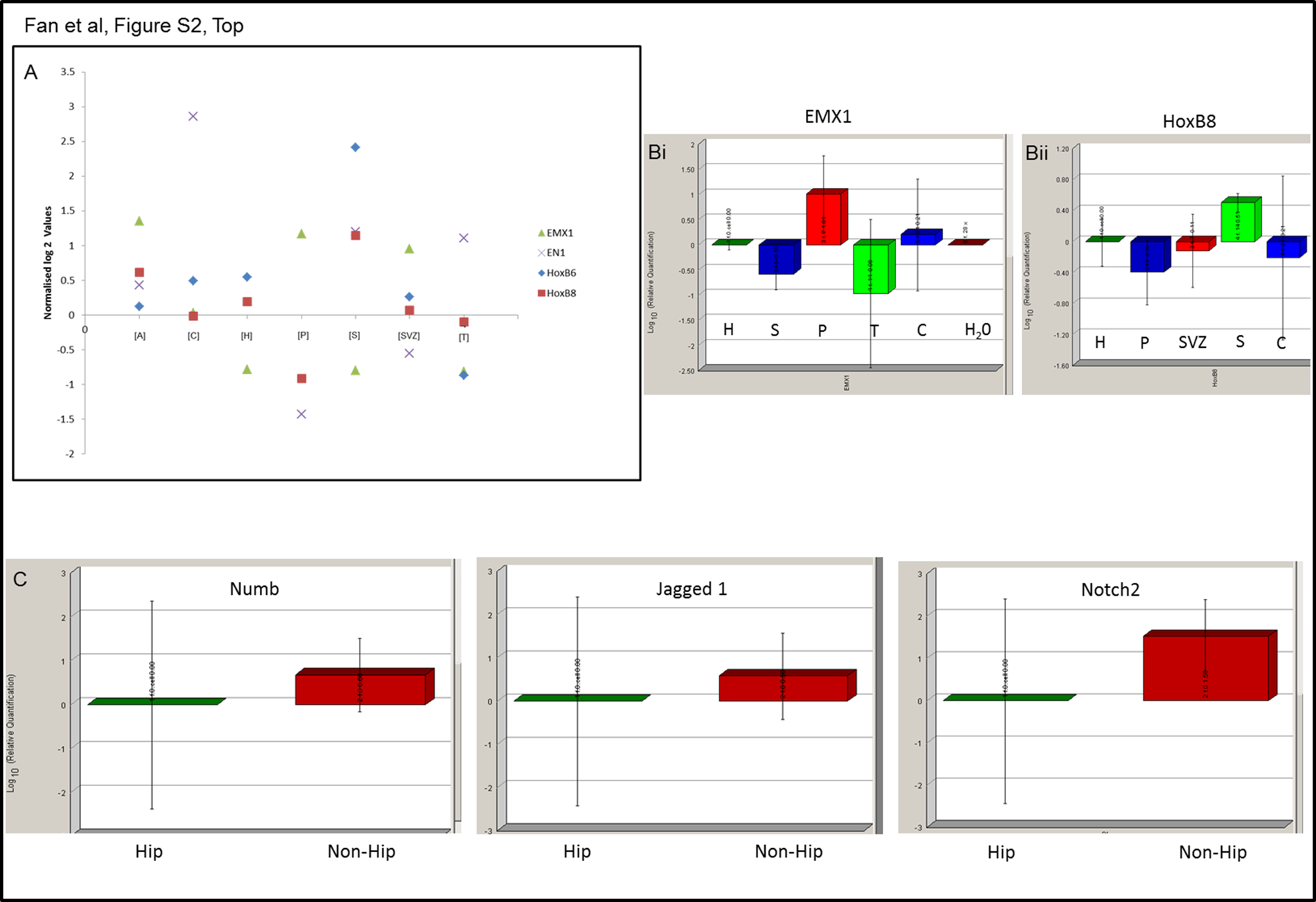

Supplement: Figure S2 — Expression levels of genes in different regions of the brain. Gene expression of region specific genes, EMX1 (cortex), EN1 (cerebellar), HoxB6 and HoxB8 (spinal cord) from the microarray (A) with EMX1(Bi) and HoxB8(Bii) corroborated by qPCR. Gene expression of numb, Jagged1 and notch2 in RNA derived from hippocampal and non-hippocampal regions by qPCR (C). The level of expression of numb (RQ:4.8), Jagged 1(RQ:8.91) and notch 2 (RQ:33.8) are higher in the non-hippocampal region. (TIF) [file pone.0105985.s002.tif]
