# Supplementary material for: Regionally-Specified Second Trimester Fetal Neural Stem Cells Reveals Differential Neurogenic Programming
Source: PLoS One. 2014 Sep 2;9(9):e105985. doi: 10.1371/journal.pone.0105985 (PMC4152177; doi:10.1371/journal.pone.0105985)
Supplement: Table S1 — Table showing the median and range of neurospheres formed per million of regionally-derived cells seeded. (DOCX) [file pone.0105985.s003.docx]

**TABLE S1 Table showing the median and range of neurospheres formed per million of regionally-derived cells seeded.**

| **Region** | **Sample (Gestation)** | **Median (spheres per million cells)** | **Range** |
| --- | --- | --- | --- |
| SVZ | S(14^+6^) | 644 | 558-656 |
|  | S(17) | 1110 | 336-1180 |
|  | S(20) | 888 | 759-924 |
|  | S(20^+3^) | 1796 | 1496-1992 |
|  | S(23^+1^) | 548 | 524-620 |
| Hippocampus | S(14+6) | 348 | 338-822 |
|  | S(17) | 404 | 332-438 |
|  | S(20) | 2024 | 1641-2377 |
|  | S(20^+3^) | 816 | 484-1028 |
|  | S(23^+1^) | 86 | 70-100 |
| Ant. Cerebrum | S(14^+6^) | 692 | 596-788 |
|  | S(17) | 424 | 272-644 |
|  | S(20) | 221 | 218-469 |
|  | S(20^+3^) | 426 | 372-480 |
|  | S(23^+1^) | 632 | 392-952 |
| Post. Cerebrum | S(14^+6^) | 488 | 386-614 |
|  | S(17) | 762 | 348-1360 |
|  | S(20) | 1166 | 952-1426 |
|  | S(20^+3^) | 844 | 720-968 |
|  | S(23^+1^) | 592 | 452-620 |
| Thalamus | S(14^+6^) | 60 | 60-340 |
|  | S(17) | 2080 | 1640-2200 |
|  | S(20) | 57.5 | 47-68 |
|  | S(20^+3^) | inadequate cell numbers | |
|  | S(23^+1^) | 588 | 462-638 |
